# Cellular and Transcriptional Responses of Resistant and Susceptible Cultivars of Alfalfa to the Root Lesion Nematode, *Pratylenchus penetrans*

**DOI:** 10.3389/fpls.2019.00971

**Published:** 2019-07-31

**Authors:** Paulo Vieira, Joseph Mowery, Jonathan D. Eisenback, Jonathan Shao, Lev G. Nemchinov

**Affiliations:** ^1^Molecular Plant Pathology Laboratory, United States Department of Agriculture – Agricultural Research Service, Beltsville, MD, United States; ^2^School of Plant and Environmental Science, Virginia Tech, Blacksburg, VA, United States; ^3^Electron and Confocal Microscopy Unit, United States Department of Agriculture – Agricultural Research Service, Beltsville, MD, United States

**Keywords:** alfalfa, *Medicago sativa*, Pratylenchidae, tannin-like deposits, transcriptome, resistance

## Abstract

The root lesion nematode (RLN), *Pratylenchus penetrans*, is a migratory species that attacks a broad range of crops, including alfalfa. High levels of infection can reduce alfalfa forage yields and lead to decreased cold tolerance. Currently, there are no commercially certified varieties with RLN resistance. Little information on molecular interactions between alfalfa and *P. penetrans*, that would shed light on mechanisms of alfalfa resistance to RLN, is available. To advance our understanding of the host–pathogen interactions and to gain biological insights into the genetics and genomics of host resistance to RLN, we performed a comprehensive assessment of resistant and susceptible interactions of alfalfa with *P. penetrans* that included root penetration studies, ultrastructural observations, and global gene expression profiling of host plants and the nematode. Several gene-candidates associated with alfalfa resistance to *P. penetrans* and nematode parasitism genes encoding nematode effector proteins were identified for potential use in alfalfa breeding programs or development of new nematicides. We propose that preformed or constitutive defenses, such as significant accumulation of tannin-like deposits in root cells of the resistant cultivar, could be a key to nematode resistance, at least for the specific case of alfalfa-*P. penetrans* interaction.

## Introduction

Alfalfa (*Medicago sativa* L.) has recently become the third most valuable field crop in the United States of America, with an estimated worth of over $9.3 billion, $1.2 billion more than wheat, according to the National Alfalfa and Forage Alliance (NAFA^1^). Nematodes are one of the major limiting factors in alfalfa production, inflicting significant damage to the plants (Westerdahl and Frate, 2008). Alfalfa is a host for several agriculturally important nematode species including the root lesion nematode (RLN), *Pratylenchus penetrans*, a migratory endoparasitic species that attacks a broad range of crops (Hafez et al., 2006; Jones et al., 2013). *Pratylenchus* spp. feed and migrate within the root cortical tissue causing a reduction in root growth after infection, accompanied by the formation of lesions, necrotic areas, browning, and cell death (Fosu-Nyarko and Jones, 2016) often followed by root rotting from secondary attack by soil fungi (Rotenberg et al., 2004) or bacteria (Vrain and Copeman, 1987). In alfalfa, high levels of infection with RLN can reduce forage yields and lead to decreased cold tolerance (Baldridge et al., 1998; Samac et al., 2015). Under severe infestation, young plants often die, because the plant’s ability to take up water and nutrients is reduced (Fosu-Nyarko and Jones, 2016; Westerdahl et al., 2017).

Although several alfalfa cultivars have been evaluated for their reaction to *P. penetrans* (Hafez et al., 2006), currently there are no commercially-certified varieties with RLN resistance. Chemical control is not a sustainable option due to the increase in production costs and negative effects on the environment. Little information on the molecular interactions between alfalfa and *P. penetrans*, that would shed light on mechanisms of alfalfa resistance to RLN, is available. The subject was last assessed in 1998 by northern blot analysis, an assay that can track only individual transcripts (Baldridge et al., 1998). Higher constitutive levels of transcripts for a few key enzymes involved in biosynthesis of isoflavonoid phytoalexins, which are implicated in resistance to both sedentary and migratory nematodes, were found in resistant alfalfa plants (Baldridge et al., 1998), suggesting that constitutive, rather than inducible, expression of defense-related genes confers enhanced resistance. Constitutively active defense responses in plants are preferred over inducible ones when there is a high probability of fast and severe pathogen attacks (Ali et al., 2012). While general processes of RLN interaction with alfalfa root cells are likely similar to those of other hosts (Fosu-Nyarko and Jones, 2016), defense reactions driven by unique transcriptional patterns of defense-related genes are expected to be species-specific, particularly in terms of resistance and susceptibility to RLN.

No single dominant natural resistance gene (*R* gene) against RLNs has been identified so far in any crop or wild plant (Fosu-Nyarko and Jones, 2016), although a few *loci* have been linked to resistance/tolerance to some RLN species in wheat (*Triticum aestivum* L.) (Jayatilake et al., 2013) or barley (*Hordeum vulgare* L.) (Sharma et al., 2011). This ultimately raises the question of the type of alfalfa resistance to *P. penetrans*: vertical, based on a single disease-resistance gene, or horizontal (polygenic) (Dyakov, 2007; Maramorosch and Loebenstein, 2009). Interestingly, the mechanism of alfalfa resistance response to other plant-parasitic nematodes (PPNs), such as the root-knot nematode (RKN), *Meloidogyne* spp., appears to be distinct: whereas a possible role of specific resistance genes was suggested for *M. sativa* and its close relative, *Medicago truncatula* Gaertn., no characteristic hypersensitive response (HR) was observed in resistant lines (Potenza et al., 2001; Dhandaydham et al., 2008). The parasitism strategy of RLN and RKN is quite different, although both can cause massive damage in alfalfa. Root-knot nematodes are considered more specialized PPNs, because they induce an exclusive feeding site (i.e., giant cells) in the vascular cylinder, from which they feed until completion of their life cycle (Moens et al., 2009). Whether or not alfalfa plants employ different or related defensive strategies against RKN and RLN, is unclear. These strategies may rely on *R* gene-mediated responses or on the products of other genes (Postnikova et al., 2015).

As in other host species, successful infection of *P. penetrans* depends on the secretion of a repertoire of proteins with diverse parasitism-related functions, such as the penetration and invasion of the host, and establishment of the nematode (Mitchum et al., 2013; Vieira et al., 2018). Identification and characterization of parasitism genes encoding nematode effector proteins and elucidating their roles in the infection process is also critical for understanding the host genetic resistance and for the development of control strategies for RLN. About 24,000 transcripts from *P. penetrans*, of which 50–55% are annotated, are currently available as a reference for transcriptome analyses (Vieira et al., 2015).

In this work, we performed a cross-level examination of resistant and susceptible interactions of alfalfa with *P. penetrans*. Differential visual, microscopic, ultrastructural, and molecular responses between a susceptible and resistant cultivar following interaction with *P. penetrans* are reported. Gene-candidates associated with alfalfa resistance to *P. penetrans* and nematode parasitism genes were identified for potential use in alfalfa breeding programs and for the development of new nematicides. This study further advances understanding of the RLN-host-interactions and, for the first time, provides biological insights into genomics of resistance of alfalfa to RLN.

## Materials and Methods

### Plant Materials

Two alfalfa checked cultivars described as resistant and susceptible (Barnes et al., 2004) were used in this study: cv. Baker (susceptible) and cv. MNGRN-16 (resistant), which supports about 60% fewer *P. penetrans* than cv. Baker. Cultivar MNGRN-16 is a 13-clone synthetic selected for vigorous shoot and root growth characteristics in the presence of large populations of *P. penetrans* at Grand Rapids, MN, United States (Petersen et al., 1991). Seeds were acquired from the National Plant Germplasm System through the Germplasm Resources Information Network (GRIN), USDA-ARS. Prior to the main experiment, both cultivars were additionally validated for their resistance/susceptibility in pilot nematode infection assays. For germination on the medium, seeds were scarified in concentrated H_2_SO_4_ for 5 min, surface sterilized with 70% ethanol for 3 min and with 1.2% sodium hypochlorite solution for 10 min, rinsed with distilled water, and placed on 1% water agar, at pH of 5.7.

### Nematode Inoculation Assays

Seven-day old alfalfa seedlings were inoculated with *P. penetrans* inoculum. *P. penetrans* isolate NL 10p RH collected from Beltsville (MD, United States) and routinely multiplied *in vitro* in ex-roots of corn (*Zea mays* L., cv. Iochief), growing in Murashige and Skoog (MS) medium agar plates, was used as inoculum. Nematodes were re-cultured every 2 months onto new ex-roots of corn and maintained in the dark at 25°C. Nematodes were prepared and plant inoculation performed as described by Vieira et al. (2017). Briefly, nematodes were extracted by placing infected roots on a wire sieve in a sterilized glass bowl filled with distilled water containing 50 mg/L carbenicillin and 50 mg/L kanamycin. After 3 days, the sieve was removed and the solution containing the nematodes poured into a 50-ml Falcon tube and centrifuged for 4 min at 4,000 *g* and 4°C. The supernatant was removed with a sterile 10-ml pipette, and the nematode pellet re-suspended with sterilized water containing both antibiotics. Alfalfa roots of both cultivars were inoculated with approximately 500 sterile nematodes (all stages). Non-inoculated plants of each cultivar grown under the same conditions served as a control. The nematode infection process and root lesion disease development were followed either macroscopically or by light microscopy from 0 to 3, 7, 21 days and 4 months after nematode infection.

### Acid Fuchsin Staining of Alfalfa Roots

To follow *P. penetrans* penetration, migration, and reproduction within infected plants, alfalfa roots were stained with acid fuchsin according to Byrd et al. (1983). Root tissues were de-stained using a clearing solution (equal volumes of lactic acid, glycerol, and distilled water) for 2 to 4 h at room temperature. After rinsing several times with tap water, roots containing nematodes were stored in acidified glycerol (five drops of 1.0M HCl in 50 ml of glycerol), and observed using a Nikon Eclipse 50i light microscope.

### Transmission Electron Microscopy

For transmission electron microscopy (TEM) analyses, nematode-infected alfalfa roots were processed as described in Vieira et al. (2017). Briefly, roots from agar cultures were dissected in fixative into 1-mm pieces, and placed under vacuum for 30 min. Tissue was fixed for 2 h at room temperature in 2.5% glutaraldehyde, 0.05M sodium cacodylate, 0.005M CaCl_2_ (pH 7.0), and refrigerated at 4°C overnight. Tissues were rinsed six times with 0.05M sodium cacodylate, 0.005M CaCl_2_ buffer, and post-fixed in 1% buffered osmium tetroxide for 2 h at room temperature. The tissues were rinsed six times in the same buffer, dehydrated in a graded ethanol series, followed by two changes of propylene oxide, infiltrated in a graded series of LX-112 resin/propylene oxide, and polymerized in LX-112 resin at 45°C for 18 h, and raised to 65°C for 24 h. Silver-gold sections of 60 to 90 nm were cut on a Reichert/AO Ultracut ultramicrotome with a Diatome diamond knife and mounted onto 100-mesh carbon/formvar-coated copper grids or onto oval slot grids. Grids were stained with 4% uranyl acetate for 10 min and 3% lead citrate for 5 min, and imaged at 80 kV with a Hitachi HT-7700 TEM.

### RNA Sequencing Experiments

For RNA-seq experiments, roots were collected at seven days after inoculation (DAI), thoroughly rinsed with distilled water, and used for total RNA extraction. Five biological replications (12–15 pooled alfalfa plants represented one replicate) were used for inoculated and non-inoculated plants of each cultivar. Total RNA extraction was performed with the RNeasy Plant Mini Kit (Qiagen) following manufacturer’s instructions. Purity and quantity of the samples were checked with a NanoDrop spectrophotometer (Thermo Scientific, United States). RNA sequencing was performed by LC Sciences (Houston, TX, United States) for a fee. cDNA libraries were generated using a poly (A) selection method and paired-end reads (2 bp × 150 bp) obtained on the Illumina HiSeq 2500 Platform.

### Read Mapping, Quantification, and Functional Analysis

To achieve a more comprehensive representation of the transcriptome, the entire pool of transcripts, including coding sequences (CDSs) and isoforms, was used to perform mapping (Zhang et al., 2016). Much of the computational analysis was performed on ARS-SCINet, a high-performance computing cluster, and a local Linux Server running RHEL 7. The whole genome sequence of cultivated alfalfa at the diploid level (CADL, 2*n* = 2*x* = 16; CADL_HM342.v0.95P) was acquired from the Medicago HapMap project^2^ and putative gene predictions were made using the GeneMark.hmm^3^ gene prediction tool (Lomsadze et al., 2005, 2014; Ter-Hovhannisyan et al., 2008). Transcripts were generated using the StringTie, and Ballgown suite using reads that were mapped by HISAT2 to the alfalfa genome. The gene calls from GeneMark.hmm and the transcripts from StringTie were merged (removing duplication) to reconstruct a comprehensive transcriptome using gffcompare^4^ (Frazee et al., 2015; Pertea et al., 2016). Gene annotation was based on BLASTX hits with the *M. truncatula* genome database downloaded from the NCBI Genbank^5^, and the NR database at NCBI. The *M. truncatula* IDs (Medtr) were assigned using the BLASTX program with Mt4.0, downloaded from the *Medicago truncatula* Genome Database. The paired-end reads were pruned for quality and adaptors using BBDuk from the BBtools software suite^6^. Alignments to the CDSs and novel transcripts of the paired-end reads were performed using HISAT2 version 2.1.0 (Kim et al., 2015). Raw counts were extracted using pileup.sh from the BBtools software suite. The DESeq2 package from Bioconductor (Anders and Huber, 2010) in the R statistics suite (R version 3.4.0) was used to estimate sample quality and expression level of the genes. The DESeq2 program performs normalization by calculating a size factor using geometric mean and median. For each comparison, the geometric mean was calculated for each gene across all samples. The counts for a gene in each sample was divided by the geometric mean. The median of these ratios in a sample was the calculated size factor for that sample. Next, this size factor was used to correct for library size or sampling depth and the composition bias of the RNA sample. After size factors were calculated to normalize the data, the estimate of dispersion was determined. DESeq2 then uses a negative binomial GLM fitting and Wald statistic in the determination of differentially expressed transcripts (DETs), where the *p*-value from the Wald Test indicates the probability that the observed difference between treatment and control was real. The adjusted value (*p*-adj) was calculated using the Benjamin–Hochberg correction. The *p*-adjusted value is similar to the false discovery rate (FDR). The *p*-adjusted value < 0.05 is the preferred value used in determining statistically-significant deferentially expressed genes. Genes with fold change more than 2, FDR less than 0.05, and number of mapped reads more than 50 were counted as differentially expressed. Similar to the procedure above, DETs of *P. penetrans* were determined by mapping paired-end reads that did not map to the alfalfa genome to the transcriptome of the same nematode isolate generated previously (Vieira et al., 2015). Since the presence of nematode RNA was low, the RPKM (Reads Per Kilobase Per Million) value was calculated for each DETs. The Blast2GO tool was used for functional categorization of DETs (Gotz et al., 2008) as previously described (Nemchinov et al., 2017). The distribution scores in the Gene Ontology (GO) charts represent the sum of sequences directly or indirectly associated to a given GO category weighted by the distance of the category to the term of “direct annotation” (User Manual, Blast2Go^7^). GO enrichment analyses were performed using the online tool agriGO (Tian et al., 2017) version 2.0^8^ with *M. truncatula* V4.0 assembly release as the reference set (Tang et al., 2014). The significantly GO enrichment terms were detected by means of the Fisher’s exact test (FDR < 0.05). Identification of transcription factors (TFs) and *R-*genes was performed using the Plant Transcription Factor Database^9^, or by manual annotation against the BLAST hits to *M. truncatula*, respectively.

### Verification of Transcriptome Data

Quantitative real-time PCR (qPCR) was performed with arbitrarily selected genes to validate expression of randomly-selected transcripts. Primers were designed using the online Realtime PCR tool (Integrated DNA Technologies, Inc., San Diego, CA, United States^10^) and alfalfa sequences generated in this work. cDNA for qPCR analyses was made using the SuperScript^TM^ III First-Strand Synthesis System with oligo d(T) (Thermo Fisher Scientific) and the same RNA samples that were used for RNA sequencing. Amplification was conducted with a CFX96 Real-time system machine (Bio-Rad), with three biological replicates using the following parameters: 95°C for 10 min (one cycle), 95°C for 10 s and 60°C for 45 s (40 cycles). The Delta Delta C(T) method (2^-ΔΔCT^) was used for analysis of relative expression (Livak and Schmittgen, 2001). The reference gene for alfalfa in all qPCR experiments was NP_001237047, a gene of unknown function with little variation in expression levels (Postnikova et al., 2013). For the qPCRs of *P. penetrans* transcripts, the *18S* rDNA gene was used as reference (Vieira et al., 2018).

## Results

### Nematode Penetration and Symptom Development in Susceptible and Resistant Cultivars of Alfalfa

**FIGURE 1:**
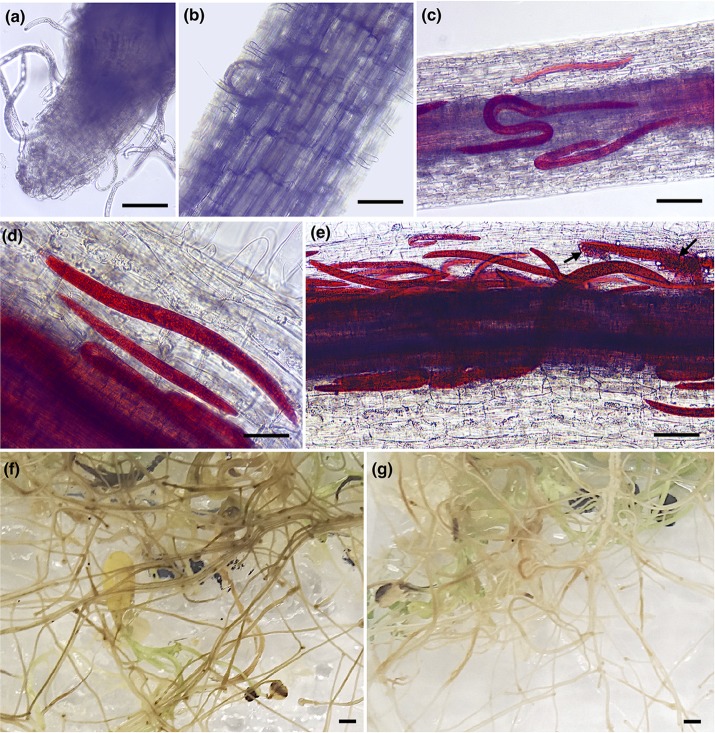
*Pratylenchus penetrans* infection progress of individual roots of susceptible (cv. Baker) and resistant (cv. MNGRN-16) alfalfa cultivars. **(a,b**) Nematodes probing and feeding on the roots of cv. Baker at 1 day after nematode infection (DAI). Acid fuchsin staining of nematode-infected root of cv. MNGRN-16 **(c)** and cv. Baker at 3 DAI **(d)**, and cv. MNGRN-16 at 7 DAI **(e)**. Eggs in **(e)** are indicated by arrows. **(f,g)** Symptoms caused by *P. penetrans* on roots of cv. Baker **(f)** and cv. MNGRN-16 **(g)** 4 months after inoculation. Root damage was found to be more severe, with larger and continuous brown lesions in the susceptible cv. Baker. Scale bars: **(a–c)**: 200 μm; **(d,e)**: 50 μm; **(f,g)**: 5 mm.

To evaluate the early infection process of *P. penetrans* in two different alfalfa cultivars, we monitored symptoms and nematode penetration in individual roots within a 7-day period after inoculation. One-day after nematode infection, all motile stages were found probing or feeding ectoparasitically on the roots of both alfalfa cultivars, with nematodes associated with the root tip or dispersed along different areas of the main infected root (Figure 1a,b). Three-days after infection nematodes were feeding mainly ectoparasitically, while some individuals had penetrated the epidermis and were distributed within the first layers of the cortex in both cultivars (Figure 1c,d). At 7 DAI, we observed a higher number of nematodes within the roots of each individual plant of both cultivars, including the deposition of eggs by females within the cortical layers of the roots (Figure 1e). The counts of individual nematodes were quantified at 7 DAI using a minimum of 10 plants in four independent biological replicates (Supplementary Figure S1). The total number of established nematodes within the roots of each individual plant ranged from 5 to 86 for the cv. Baker, and 3 to 88 nematodes for the cv. MNGRN-16, suggesting a high variability in the infectivity of the nematodes on the different plants at the 7 DAI. To follow up on the infection process on later stages of the infection and to evaluate phenotypic differences between cultivars in response to *P. penetrans*, we performed *in vitro* nematode assays 4 months after inoculation. Root damage was more severe, with larger and continuous lesions in the cv. Baker than in the roots of cv. MNGRN-16 (Figure 1f,g). Non-infected roots of both cultivars showed no lesions and displayed a typical healthy phenotype (data not shown). Therefore, despite the earlier signs of the infection process appeared essentially identical in the susceptible and resistant cultivars, it may be assumed that these initial host–pathogen interactions, while of visibly susceptible type, could nevertheless be important for the forthcoming inhibition of the infection in the resistant line. In other words, any mechanism of resistance in alfalfa to the RLN used is not obvious in the changes of the root in the first 7 DAI.

### Ultrastructural Observations

To explore the subcellular changes in the roots of both alfalfa cultivars in response to *P. penetrans*, we performed TEM of non-infected and nematode-infected roots at 7 DAI (early time point) and 4-months (late time point) after nematode infection.

**FIGURE 2:**
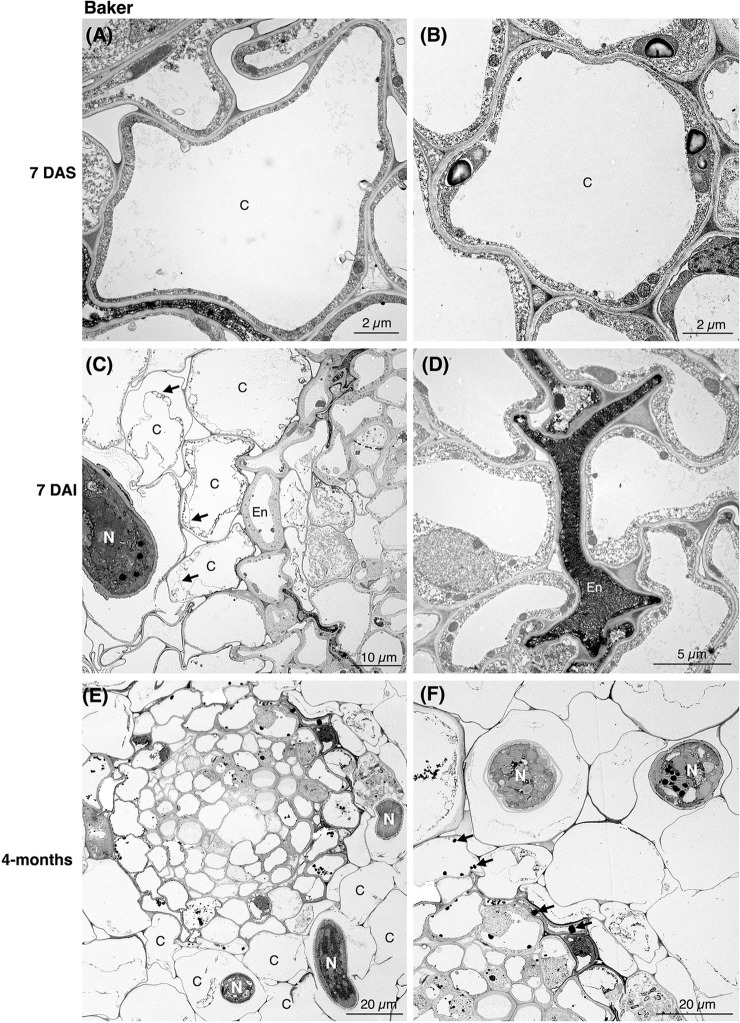
Transmission electron microscopy images of non- and nematode-infected root cells of susceptible alfalfa cv. Baker. **(A,B)** Control (non-infected) cortical cells at 7 days after seedling present no accumulation of tannins. **(C,D)** Nematode-infected roots at 7 days after infection (DAI) presented cortical cell devoid or with disrupted cytoplasm (arrows in **C**). Cortical and endodermal cells adjacent to the nematode often displayed a compressed phenotype **(D)**, and accumulation of tannin-like deposits (TLDs). **(E,F)** 4-month after nematode infection a more pronounced damage of the cortical cells could be observe by the nematode activity **(E)**, with randomly accumulation of TLDs of the cells adjacent to the nematode (arrows in **D**). C, cortex cell; En, endodermis; N, nematode.

In the susceptible cv. Baker, the epidermal cells at 7 DAI were already affected by the nematode activity. As nematodes fed and progressed into the inner cells of the cortex, the cells became devoid of cytoplasmic content and lost their membrane integrity (Figure 2C). During nematode migration, cavities between the cells resulting from the breakdown of continuous cortical parenchyma cells were formed. In some cells of cv. Baker the accumulation of electron-dense, tannin-like deposits (TLDs) randomly occurred, noticeably varying among the different cells adjacent to the sites of nematode activity, while in control roots no TLDs were detected (Figure 2A,B). The endodermal cells were often collapsed, displaying a dense and dark cytoplasm with the accumulation of TLDs (Figure 2D). At 4-months after inoculation, extensive damage to the cortex cells of the roots of cv. Baker was observed, as a higher number of nematodes parasitizing the roots could be seen in cross sections of the cortex (Figure 2E,F). The symptoms observed at this time point were more severe compared to those observed at 7 DAI, and thus consistent with visual or macroscopic observations.

**FIGURE 3:**
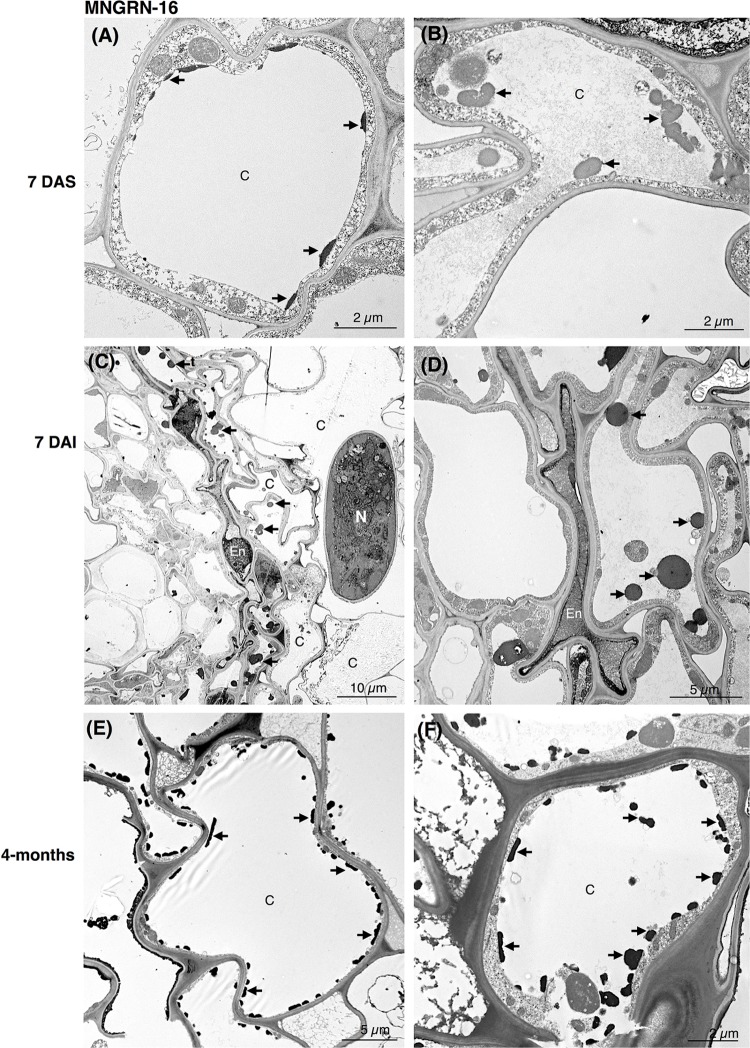
Transmission electron microscopy images of non- and nematode-infected root cells of resistant alfalfa cv. MNGRN-16. **(A,B)** Control (non-infected) cortical cells at 7 days after seedling presenting constitutive accumulation of tannin-like deposits (TLDs). **(C,D)** Nematode-infected roots at 7 DAI presented cortical cell destruction due to the nematode activity. Both cortical and endodermal cells presented a high accumulation of tannin-like deposits (TLDs) indicated with arrows. **(D)** Endodermal cells adjacent to the nematode often displayed a compressed phenotype. **(E,F)** 4-month after nematode infection the cortical cells presented a generalized distribution of TLDs, in both the cytoplasm and vacuole. C, cortex cell; En, endodermis; N, nematode.

Ultrastructural studies of the resistant cv. MNGRN-16 also revealed some destruction of the epidermal and cortical cells after nematode penetration into the roots, resembling to a certain level those in cv. Baker (Figure 3). However, root cells of cv. MNGRN-16 differed strikingly in the accumulation of TLDs, displaying consistently higher numbers of large TLDs widely dispersed in all the cortex cells (Figure 3C,D). Characteristically, non-infected roots of cv. MNGRN-16 also contained a large number of TLDs, suggesting their constitutive presence and formation in the resistant cultivar prior to nematode infection (Figure 3A,B). Four months after infection, distribution of nematodes within the roots of cv. MNGRN-16 was patchy and often difficult to visualize and less root lesions could be observed at this time point. Although some cell wall damage of the cortical cells could be identified, the level was less intense than in the cv. Baker (Figure 3E,F). A significant accumulation of TLDs distributed in all the cortical cells was still observed. In summary, the TEM observations confirmed root penetration studies, pointing to the similarities between the cultivars in their early responses to nematode infection and considerable differences later in the infection cycle. Along with the significantly less damage observed 4 months after nematode inoculation in the root cells of the resistant line, their most distinctive ultrastructural feature compared to the susceptible cultivar was a substantial accumulation of TLDs in both non-infected and nematode-infected roots.

### Transcriptome Profiling of Resistant and Susceptible Host Responses

#### Metrics of RNA-Seq Data

A total of 1,008,407,732 pair-end reads were generated from 20 cDNA libraries, averaging 50,420,386 reads per library (Supplementary Table S1). An alignment rate for each library ranged between 64 and 76% of all reads mapped to the reference CADL genome. Overall, the data obtained by mRNA-seq were considered to be sufficient for host gene expression profiling, as well as for profiling of the RLN genes in resistant and susceptible alfalfa cultivars.

The following hypotheses were tested: (1) host defense responses will be up-regulated in response to nematode infection in resistant plants; and (2) host defense responses in susceptible plants will be suppressed and/or otherwise different from resistant plants upon infection with nematodes; (3) *P. penetrans* will develop specific adaptive responses to conditions imposed by different types of plant immune system.

### Differentially Expressed Transcripts (DETs) in the Resistant Alfalfa Cultivar MNGRN-16

Total counts of transcripts, differentially expressed in the nematode-infected plants of cv. MNGRN-16, are shown in Table 1A and Supplementary Table S2.1. Genes expressed only in the infected plants of cv. MNGRN-16 (unique genes) are also presented (Table 1A and Supplementary Table S2.2). A few general observations were made regarding the numbers, composition, and representation of these DETs: (1) DETs counts were low; (2) counts of up- and down-regulated DETs were similar (3) nematode-infected plants had several up-regulated genes with possible roles in plant defense responses.

**Table 1:** Differentially expressed transcripts found between each intra-cultivar **(A)** and inter-cultivar **(B)** interaction.

(A) Intra-cultivar comparisons						
**MNGRN-16 RLN/Control**				**Baker RLN/Control**		

	**Up**	**Down**	**Total**	**Up**	**Down**	**Total**

Transcripts	18 (2)^∗^	15 (8)	33	175 (32)	26 (3)	201

**(B) Inter-cultivar comparisons**						

**MNGRN-16 Control/Baker Control**				**MNGRN-16 RLN/Baker RLN**		

	**Up**	**Down**	**Total**	**Up**	**Down**	**Total**

Transcripts	2,529 (99)	2,203 (150)	4,732	1,630 (58)	1,313 (115)	2,943


*^∗^Values between parentheses represent the number of unique genes.*

Low counts of DETs in plants infected with migratory nematodes are not unusual. For example, Yu et al. (2015) found 137 genes that exhibited different transcript abundances between two libraries derived from control and experimental ramie plants, infected with RLN *Pratylenchus coffeae*. Similarly, low number of DETs was reported in rice roots infected with the root rot nematode, *Hirschmanniella oryzae* (Kyndt et al., 2012). Just like the low counts, a similar ratio of up- and down-regulated DETs indicates a limited scale of affected host pathways and a well-coordinated, balanced host response.

A total of 16 DETs were up-regulated (>2-fold change and FDR > 0.05) in the nematode-infected alfalfa plants (Table 1A). High expression of genes encoding ubiquitous UDP-glucosyltransferase (Medtr6g042310.1) family proteins (UGTs) of the secondary metabolic pathways may not be accidental: importance of UGTs in plant–pathogen interactions was previously demonstrated (Gachon et al., 2005). Overexpression of Medtr4g033085.1, encoding isoflavone-7-*O*-methyltransferase is particularly interesting, as this gene has been implicated in disease resistance in alfalfa (He and Dixon, 2000). Besides, its overexpression could be related via phenylpropanoid and flavonoid pathways to the increased formation of condensed TLDs found in the infected plants of the resistant cultivar of alfalfa (see section “Transmission Electron Microscopy”). Roles of tannins in plant defenses against leaf-eating herbivores and pathogens are well-documented (Constabel et al., 2014). Three induced DETs, encoded receptor-like kinases, which are the components of signal reception, signaling and plant defense, and the basis for pathogen recognition and specificity of plant response. In addition, *P. penetrans*-infected plants expressed two important genes that were absent in the control libraries: (1) Medtr6g005630.1, encoding a polygalacturonase, an enzyme that is involved in degradation of pectin, a major component of plant cell walls, and (2) Medtr6g046570.1, encoding a disease resistance protein of the CC-NBS-LRR class, involved in pathogen recognition and activation of defense responses (Jones et al., 2016). While expression of polygalacturonase suggests that *P. penetrans* may possibly induce rearrangement of the cell wall architecture (Berg and Taylor, 2009), specific activation of innate pathogen detection and defense gene Medtr6g046570.1 may point to the direct role in resistance to *P. penetrans*.

Based on the low read numbers in three or more replicates, eight genes were considered to be unique to the control, non-infected plants (Table 1A and Supplementary Table S2.2). Among them were several important genes, encoding F-box protein domain, WRKY family TF and disease resistance protein Medtr3g086070.1. The latter protein was reported to be expressed in *M. truncatula* at the basal levels when compared to highly expressed genes implicated in resistance pathways (Nepal et al., 2017). Low or undetectable expression levels of these genes in nematode-infected plants suggests that they might not be involved in the resistance process against *P. penetrans*. Alternatively, their loss of function in the infected plants can contribute toward increased resistance either by way of negative regulation, as it was reported for WRKY TFs (Journot-Catalino et al., 2006) and F-box protein SON1 (Kim and Delaney, 2002), or by way of passive loss of susceptibility that is, loss of interaction with pathogen effectors (Kourelis and van der Hoorn, 2018).

Thus, three groups of genes involved in cell wall biogenesis, secondary metabolic pathways and NLR-dependent immune reactions (nucleotide-binding domain, leucine-rich repeat domain-containing proteins) (Jones et al., 2016), appear to play important roles in regulation of defense responses against *P. penetrans* in the resistant alfalfa cultivar MNGRN-16.

### Differentially Expressed Transcripts in Susceptible Alfalfa Cultivar Baker

Total counts of DETs in nematode-infected plants of the susceptible cv. Baker and genes uniquely expressed in the infected plants of this cultivar, are shown in Table 1A and Supplementary Table S3. DETs counts in the susceptible plants were not as low as in the resistant cultivar and involved 201 transcripts (including uniquely expressed genes).

The number of up-regulated DETs was sevenfold higher than that of the down-regulated transcripts (143 vs. 23), implying the larger quantity of cellular components/resources required for a compatible interaction of the susceptible cultivar with the nematode. Among up-regulated transcripts were several DETs mapped to genes related to biogenesis of the cell wall (Medtr2g090765.1 and Medtr7g075453.1), a pectinesterase inhibitor (Medtr7g050980.1), β-1,3-glucan hydrolase (Medtr5g044530.1) and a chitinase (Medtr3g118390.1) (Supplementary Table S3.1). High levels of chitinase activity have been previously reported in banana plants infected with RLN, *P. coffeae* (Backiyarani et al., 2015). Supporting our TEM results, several genes involved in the secondary metabolism pathway (4-coumarate: CoA ligase-like proteins Medtr3g088880.1 and Medtr3g088870.1; and the UDP-glucosyltransferase family protein Medtr5g019580.2) were also induced upon nematode infection. 4-coumarate:CoA ligases are a group of essential enzymes involved in the metabolism of phenylpropanoid-derived compounds, and are often associated with the production of different classes of secondary metabolites.

Among genes down-regulated in the infected plants of cv. Baker were those for receptor-like kinases (Medtr7g066590.1, Medtr1g039310.1, Medtr1g038890.1), an ortholog of the *Rpp4C4* candidate gene (Medtr8g059275.1) for resistance against Asian soybean rust (*Phakopsora pachyrhizi*) in soybean (*Glycine max*) (Meyer et al., 2009), a wound-responsive gene (Medtr5g023110.1), and a gene encoding putative GRF (growth-regulating factor) TF (Medtr5g047980.1) implicated in diverse biological processes in plants (Omidbakhshfard et al., 2015) (Supplementary Table S3.1).

Infected plants of cv. Baker uniquely expressed 32 up-regulated genes (Supplementary Table S3.2), including highly induced ortholog of the *M. truncatula* disease resistance protein (Medtr2g071820.1).

Surprisingly, when the overall responses of both cultivars to *P. penetrans* were compared, no common DETs were found in the infected plants of both cultivars, i.e., the transcription modulation in each cultivar was distinct, presuming a broad specificity of resistant and susceptible alfalfa interactions with this particular pathogen. In general, despite a higher number of genes were activated in cv. Baker upon *P. penetrans* infection, this interaction is of the susceptible type, which agrees with our cellular and molecular analyses.

### Inter-Cultivar Differences in Constitutive Gene Expression in Non-infected Plants

While internal comparisons of differential gene expression provided information on cultivar-specific responses to RLN, they did not explain disparities between the cultivars in terms of their resistance to the pathogen. For that purpose, we compared basal or constitutive gene expression between both cultivars without nematode infection and evaluated it against expression of genes, responsive to the RLN infection (Table 1B and Supplementary Table S4). This was done to reveal any differences in the constitutive gene expression between resistant and susceptible varieties that may have a critical role prior to infection, affecting subsequent susceptibility or resistance to *P. penetrans*.

To achieve this, we looked at the DETs constitutively up-regulated in cv. MNRGN-16 in non-infected plants (i.e., basal ratio MNGRN-16/Baker). Since cultivars were compared to each other, DETs up-regulated in cv. MNGRN-16 would be down-regulated in cv. Baker and vice-versa (Table 1B). There were 4,483 DETs (both up- and down-regulated) between the two control, non-nematode infected cultivars, which included 2,430 up-regulated DETs in cv. MNGRN-16. Expression of the same genes, whether repressed or induced, in two different cultivars, underlie their species-specific genome similarities (Supplementary Table S4.1). There also were unique genes expressed only in one cultivar but not in the other (99 in cv. MNGRN-16 and 150 in cv. Baker), which, in turn, is indicative of the cultivar – and/or genotype-specific differences (Table 1B and Supplementary Table S4.2).

**FIGURE 4:**
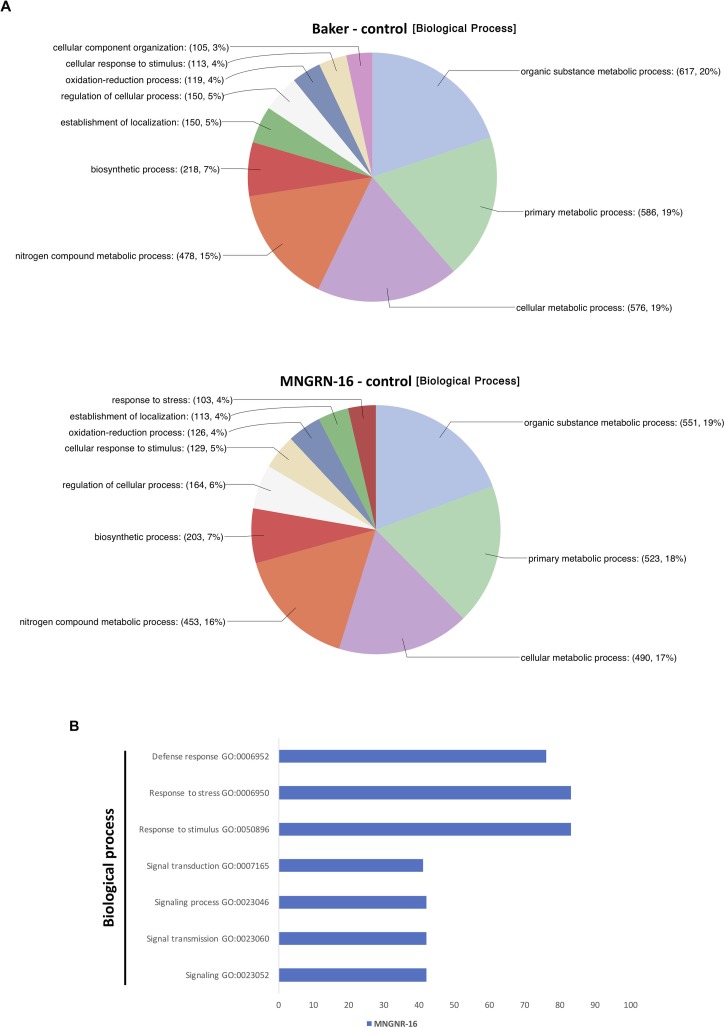
Functional characterization of up-regulated DETs. **(A)** Functional characterization of up-regulated DETs in non-infected plants of the resistant cultivar cv. MNGRN-16 and susceptible cv. Baker using GO annotation. **(B)** Functional GO enrichment analysis of the DETs sets, identified in non-infected plants of cv. Baker and cv. MNGNR-16 and performed using agriGO toolkit. The enriched GO terms (FDR < 0.05) are plotted in relation to the number of transcripts.

Functional characterization of the up-regulated genes with Blast2GO tool showed that distribution of GO categories in a key domain “biological process” was nearly identical in the two cultivars, except for the category “response to stress,” which was absent in the cv. Baker (Figure 4A). Missing of the entire category in cv. Baker may illustrate its predisposition to *P. penetrans* infection, or “an internal degree of susceptibility” (Yarwood, 1959; Schoeneweiss, 1975). Up-regulated DETs in cv. MNGRN-16 included some key genes, which respond to diverse environmental stresses, such as genes for universal stress proteins (Medtr1g083950.1 and Medtr1g087200.1), the highly-expressed adenine nucleotide alpha hydrolase superfamily protein (Medtr1g054765.1), which appears to be an ortholog of *Arabidopsis* universal stress protein At3G53990 (Melencion et al., 2017), receptor kinases (Medtr5g086040.3 and Medtr6g005210.1), defensins (Medtr8g070770.1 and Medtr2g079440.1), pathogenesis-related proteins (Medtr8g045570.1 and Medtr8g045570.1), calmodulin-binding transcription activator, involved in modulation of biotic and abiotic stress (Medtr8g090205.1) (Doherty et al., 2009), ABA/WDS-induced protein (Medtr6g037220.1) (Supplementary Table S4.3).

Gene Ontology enrichment analysis with the same datasets of DETs up-regulated in control plants of MNGRN-16 and Baker cultivars confirmed the results of functional GO categorization, i.e., the DETs set of cv. MNGRN-16 was enriched in the GO terms related to the category “biological process” including those related to stress responses (Figure 4B and Supplementary Table S4.4). GO enrichment analysis of the DETs up-regulated in cv. Baker did not identify any enriched GO terms in the category “biological process” (FDR < 0.05) that were significantly over-represented in this gene set (Supplementary Table S4.4).

One hundred and seventy of the DETs that were up-regulated in the resistant cultivar MNGRN-16 putatively encoded NLR disease resistance proteins (Supplementary Table S4.5). Forty-six of them, encoding disease resistance proteins of the TIR-NBS-LRR class, were identified by Blast2GO as potentially involved in response to stimulus (Supplementary Table S4.3). Thirteen of the up-regulated transcripts (Medtr8g028500.1) were orthologs of the *Rpp4* (*Rpp4C1* and *Rpp4C4*) candidate genes for resistance against Asian soybean rust in soybean (Meyer et al., 2009). In cv. Baker, only a single *Rpp4C4* was up-regulated. A cluster of nine up-regulated *R* genes containing three putative orthologs of *RGA4* gene, that was proposed to trigger an *avr* (avirulence gene)-independent cell death in rice and *Nicotiana benthamiana* (Cesari et al., 2014), was found only in the resistant cultivar (unique genes).

More than 50 DETs that mapped to putative TFs were constitutively induced in the cv. MNGRN-16 and were mostly of the WRKY and MYB families (10 and 13, respectively). Among the TFs with elevated expression levels were representatives of other major TF families, including the AP2/ERF, plant-specific YABBY TFs, BZIP, bHLH, NAC (Supplementary Table S4.6).

Biosynthetic pathway of proanthocyanidins (condensed tannins) formation includes several key enzymes, such as flavanone 3-hydroxylase (F3H); dihydroflavonol reductase; leucoanthocyanidin reductase (LAR); anthocyanidin synthase (ANS), and anthocyanidin reductase (ANR) (Dixon et al., 2005). One of those genes (F3H, Medtr5g059140.1) was up-regulated in cv. MNGRN-16, but there was no expression in cv. Baker. Cultivar MNGRN-16 also had eight highly (log 2-fold change > 2) up-regulated genes encoding aldo–keto reductase proteins (AKRs) that are predominantly involved in the plant secondary metabolic pathways, including flavonoid biosynthesis (Sengupta et al., 2015).

Ninety nine unique genes were induced only in the resistant cultivar (Table 1B), and these included a cluster of nine *R* genes mentioned above; a group of trans-membrane proteins (10), reported to play essential roles in sensing and response to environmental stresses; subtilisin-like serine endopeptidase (Medtr5g081100.1), presumably involved in plant–pathogen recognition and immune priming (Figueiredo et al., 2014); nonsense-mediated mRNA decay (NMD) protein (Medtr5g006990.1) of the quality-control mechanism that contributes to plant defenses (Shaul, 2015); flavin-containing monooxygenase (Medtr7g099160.1), an ortholog of the *Arabidopsis* YUC genes (Di et al., 2016) (Supplementary Table S4.2). Up-regulation of the latter gene (Medtr7g099160.1), which is linked to auxin biosynthesis and overproduction phenotype, demonstrates that auxin signaling pathway may be among those critical for the state of “increased alertness” in resistant plants before exposure to the pathogen. The role of auxin in plant–pathogen interactions is widely acknowledged (Kazan and Manners, 2009).

Taken together, these results suggest that constitutive gene expression in two cultivars differs significantly and could be a reason behind subsequent susceptibility or resistance to *P. penetrans*.

### Inter-Cultivar Differences in Gene Expression Under RLN Infection

We have followed up on the analysis of DETs between the resistant and the susceptible cultivars in response to nematode infection (Table 1B and Supplementary Tables S5.1, S5.2). The number of up-regulated transcripts of AKRs in the infected cv. MNGRN-16 remained high as in non-infected plants, with many of them expressed by more than four-fold. Enzymes of the AKR family play multiple roles in antioxidant defenses in plants, are involved in detoxification of stress-induced reactive carbonyls and represent a potential target for the development of stress-tolerant plants (Sengupta et al., 2015). F3H (Medtr5g059140.1, flavanone 3-hydroxylase), constitutively up-regulated in control plants of the resistant cultivar, was not differentially expressed in the RLN-infected plants.

The response of cv. MNGRN-16 to *P. penetrans* also included activation of unique genes belonging to the secondary metabolism pathways, such as 4-hydroxy-3-methylbut-2-enyl diphosphate reductase (Medtr4g069070.1), which catalyzes the last step of the methylerythritol phosphate pathway to synthesize isopentenyl diphosphate (IPP) and its allyl isomer dimethylallyl diphosphate. IPP is a precursor for biosynthesis of terpenoids, which can function as phytoalexins in plant direct defense (Cheng et al., 2007; Pichersky and Raguso, 2017). Induction of 2OG-Fe(II) oxygenase family oxidoreductase (Medtr7g063730.1), normally involved in plant flavonoid biosynthesis (Cheng et al., 2014), and a dihydroflavonol reductase (Medtr2g101330.1), a key enzyme in biosynthesis of anthocyanins (Wang et al., 2013), suggests a multifaceted regulation of secondary metabolites in cv. MNGRN-16 and their potential roles in the mechanism of resistance to RLN.

One hundred and twenty six DETs (a decrease from 170 in control conditions), that mapped to *R* genes, were up-regulated in the cv. MNGRN-16, and these included the orthologs of the *Rpp4C1* and *Rpp4C4* soybean genes, once again indicating their importance in resistance to RLN, both prior to infection and during the pathogen attack (Supplementary Table S5.3). The number of up-regulated genes, uniquely expressed in the infected cv. MNGRN-16, decreased nearly twofold (Supplementary Table S5.2). A similar pattern was observed for the overall number of genes expressed in both nematode-infected cultivars. Among the unique genes in cv. MNGRN-16 (not expressed in cv. Baker), two *R* genes were up-regulated under RLN infection: Medtr4g015030.1 (disease resistance protein of the TIR-NBS-LRR class, log 2-fold change = 6.84) and Medtr5g070960.1 (*RGA4* gene, log 2-fold change = 3.76). Interestingly, a gene encoding an ortholog of subtilisin-like serine endopeptidase (Medtr5g081100.1) was highly expressed (log 2-fold change = 9.93) as it was in the non-infected plants. A transcript putatively encoding F-box protein (Medtr1g015450.1) was highly expressed following the RLN infection, similarly to expression in the uninfected controls. Receptor-like membrane-anchored glycoproteins with extracellular leucine-rich repeats (LRRs), are encoded by a distinct class of *R* genes and were reported to regulate cell death and pathogen responses in infected tobacco (*Nicotiana tabacum*) and tomato (*Solanum lycopersicum*) (van den Burg et al., 2008).

**FIGURE 5:**
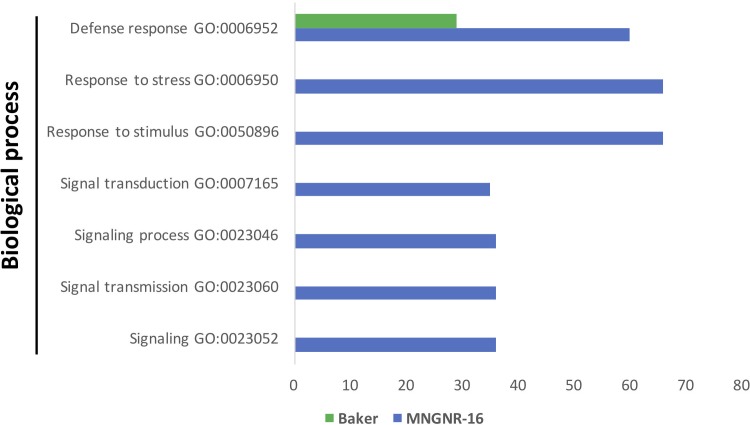
Functional GO enrichment analysis of the DETs sets, identified in nematode-infected plants of cv. Baker and cv. MNGNR-16 and performed using agriGO toolkit. The enriched GO terms (FDR < 0.05) are plotted in relation to the number of transcripts.

Gene Ontology enrichment analysis of the DETs sets up-regulated in both cultivars in response to the nematode nearly mirrored the results of the enrichment analysis performed on datasets of DETs up-regulated in the uninfected controls: the same GO terms were significantly overrepresented in cv. MNGRN-16 dataset (Figure 5 and Supplementary Table S5.4). Although enrichment analysis assigned several DETs to the GO term “defense response” in cv. Baker dataset, the number of DETs associated with this GO term was significantly less than in the cv. MNGRN-16 (Supplementary Table S5.4).

Transcription factors activity appeared to be somewhat reduced in the infected plants of cv. MNGRN-16: 41 DETs that mapped to TFs were induced during infection vs. 58 DETs in non-infected plants (Supplementary Table S5.5). Only two WRKY and nine MYB TFs were activated. Unlike in the control plants, the majority of the MYB TFs (six) contained a SANT domain that is mainly found in proteins involved in chromatin functions and often recognize histone tails (Feller et al., 2011).

### Transcriptome Profiling of Nematode Genes

To identify putative nematode transcripts within the different libraries generated from alfalfa-infected roots, all reads that did not map to the alfalfa genome were mapped to the transcriptome generated previously for the same *P. penetrans* isolate (Vieira et al., 2015). These were 547,897 and 475,043 reads from the cultivars Baker and MNGRN-16, respectively. The coverage for the nematode transcripts was on average low. For analysis, nematode transcripts were selected using the total average values of RPKM > 1. When all the libraries were compared, a total of 15,150 and 15,065 nematode transcripts were identified within the infected roots of Baker and MNGRN-16 cultivars, respectively (Supplementary Table S6.1). Only transcripts with the most reads that were represented in both libraries were considered relevant. These top transcripts encoded housekeeping, metabolic, and developmental proteins, such as ribosomal proteins, structural components of the cuticle, and products with a capacity to bind to cytoskeletal proteins, collagen, and actin. A significant number of transcripts encoded proteins of unknown functions.

When the full set of nematode transcripts was considered, most of them overlapped and the majority displayed no significant differential expression between the two cultivars. Nevertheless, 319 DETs (*p* < 0.05) were identified (Supplementary Table S6.2). To further examine potential functional significance of these DETs, GO terms were assigned to all transcripts using Blast2GO. Of the transcripts belonging to the GO class “Molecular function,” the most represented groups were involved in protein and ATP binding (Supplementary Figure S2). Remarkably, several transcripts encoding collagen proteins of the nematode cuticle were among the DETs highly expressed in the susceptible cv. Baker. These genes participate in significant biological processes of the nematode ontogenesis, such as the development of larvae, collagen trimer, and structural constituents of the cuticle. Since nematode development is punctuated by different molting stages and production of a new cuticle, these results suggest that, in spite of the fact that nematodes are able to penetrate roots of both cultivars, their development might be arrested within the resistant cv. MNGRN-16.

### Identification of *Pratylenchus penetrans* Effectors

Root lesion nematode can secrete a set of proteins that are deployed to the host–nematode interface during infection, and these secreted proteins are expected to play a central role in the nematode parasitism. To identify transcripts essential to *P. penetrans* parasitism, we looked into nematode reads that mapped to the set of transcripts encoding putative secreted proteins without transmembrane domain. First, the datasets from the alfalfa-RLN experiments were screened for candidate effector genes, previously identified in *P. penetrans*. We found that transcripts for all 22 candidate effectors, identified in the esophageal glands of *P. penetrans* (Vieira et al., 2018), were actively transcribed by the nematodes during interaction with both cultivars (Table 2), although their expression levels were variable (11 < RPKM < 1,891). This indicated that nematode reads, recovered from the infected roots, contained sufficient depth to detect candidate effector genes produced by the esophageal glands of *P. penetrans.* Interestingly, genes encoding five pioneer effector proteins identified so far in *P. penetrans* (Vieira et al., 2018), were among the highly expressed transcripts (Table 2 and Supplementary Table S6.1). Several transcripts encoding for nematode cell wall-degrading enzymes, which are often associated with the host cell wall degradation or modification, were also highly expressed. The most prominent were transcripts encoding an expansin-like protein, an endo-β-1,4-glucanase (GH5), and two pectate lyases (PL3). In addition, consistent with the previous studies, transcripts encoding for nematode effectors involved in suppression of plant defenses, such as venom allergen-like protein (Lozano-Torres et al., 2014) and a calreticulin (Jaouannet et al., 2013), were actively transcribed during nematode-alfalfa interaction, as well as other genes with unknown functions, such as FARs and SXP/RAL-2, previously detected within the esophageal glands of *P. penetrans* (Vieira et al., 2018).

**Table 2:** Relative expression (RPKM) of *Pratylenchus penetrans* candidate effectors in cv. Baker and cv. MNGNR-16, respectively.

Transcript ID	cv. Baker (RPKM)	cv. MNGNR-16 (RPKM)	Predicted protein function	E-value	Top hit species (NCBI)	Best blast hit (NCBI)
Ppen11402_c0_seq1^∗^	1891.00	1441.00	Pioneer	–	–	–
Ppen16493_c0_seq1	1703.00	1680.00	Catalase	0	*Ditylenchus destructor*	AFJ15102.1
Ppen12016_c0_seq1^∗^	826.75	1011.11	Pioneer	–	–	–
Ppen16605_c0_seq1^∗^	746.18	727.01	Pioneer	–	–	–
Ppen7984_c0_seq1^∗^	634.70	929.25	Pioneer	–	–	–
Ppen10370_c0_seq1^∗^	586.09	928.43	Pioneer	–	–	–
Ppen12533_c0_seq1	566.00	438.00	Expansin-like protein	1.60E-40	*Heterodera avenae*	APC23320.1
Ppen8004_c0_seq1^∗^	541.00	676.00	Pioneer	–	–	–
Ppen15229_c0_seq1	507.97	546.71	Calreticulin	0	*Pratylenchus goodeyi*	AIW66697.1
Ppen15842_c0_seq1	385.22	452.17	Beta-1,4-endoglucanase	0.00E+00	*Pratylenchus penetrans*	BAB68522.1
Ppen12103_c0_seq1	372.68	270.87	SXP RAL-2 protein	6.60E-34	*Meloidogyne incognita*	AAR35032.1
Ppen15554_c1_seq3	250.00	268.00	Expansin B3	4.30E-44	*Heterodera glycines*	ADL29728.1
Ppen13447_c0_seq1	202.00	329.00	Pectate lyase 2	7.70E-85	*Heterodera glycines*	ADW77534.1
Ppen14256_c0_seq1	152.20	153.59	Pectate lyase 1	2.60E-48	*Globodera pallida*	AEA08853.1
Ppen15066_c0_seq1	141.23	62.96	Pioneer	–	–	–
Ppen12895_c0_seq1	89.87	153.85	Fatty acid and retinol binding protein	7.10E-27	*Pratylenchus penetrans*	APT68073.1
Ppen13849_c0_seq1	88.59	85.45	Trypsin Inhibitor like cysteine rich domain protein	1.10E-09	*Pristionchus pacificus*	PDM81086.1
Ppen18759_c0_seq1	49.88	19.53	Arabinogalactan endo-1,4-beta-galactosidase	4.1E-123	*Heterodera schachtii*	ACY02855.1
Ppen11632_c0_seq1	42.52	70.60	Venom allergen-like	4.00E-84	*Globodera rostochiensis*	AEL16453.1
Ppen16218_c0_seq1	38.00	111.00	Beta-1,4-endoglucanase	3.20E-80	*Pratylenchus coffeae*	ABX79356.1
Ppen11230_c0_seq1^∗^	17.11	48.02	Pioneer	–	–	–
Ppen12597_c1_seq1	11.61	19.20	Glucuronoarabinoxylan endo-1,4-beta-xylanase	0.00E+00	*Radopholus similis*	ABZ78968.1


*^∗^The sequences of these genes are available from the Dryad Digital Repository: https://doi.org/10.5061/dryad.4h44313 (Vieira et al., 2018).*

Blast analyses also identified transcripts encoding orthologs of the candidate effector genes known in other PPNs (Supplementary Table S6.1), but not yet confirmed as candidate effectors in *P. penetrans*. The resulting set of transcripts contained numerous genes encoding secreted proteins with relevance to the nematode-plant interaction of both migratory and sedentary nematode species. It included those for several transthyretin-like proteins (Lin et al., 2016), different classes of peptidases, several homologs of putative effectors recently identified for *Heterodera avenae* (Chen et al., 2018), and genes related to oxidative stress and production of ROS. A significant proportion of nematode transcripts encoding putative secreted proteins and highly expressed in alfalfa had no functional annotation and thus could contain additional candidate effectors of *P. penetrans* involved in plant parasitism.

### Confirmation of Transcriptomic Data by Quantitative Real-Time PCR (qPCR)

Quantitative real-time PCR (qPCR) was performed with 31 arbitrarily selected alfalfa genes, identified as differentially expressed based on analysis of the transcriptome (Table 3). DETs were selected from all comparisons discussed above for both alfalfa cultivars. qPCR data and the corresponding RNA-seq values were comparable for 93% of the genes tested.

**Table 3:** Validation of RNA-seq data by quantitative real-time PCR.

Cultivar/ID	Annotation *Medicago truncatula*	*M. truncatula* ID	Primers	RNA-seq	qPCR
				
				Log 2-Fold Change	
**cv. MNGRN-16 control vs. cv. Baker control**					
82337_t	Isoflavone-7-O-methyltransferase	Medtr4g033085.1	LN629-630	-1.78	1.96
193689_t	Universal stress family protein	Medtr1g083950.1	LN631-632	1.16	1.75
45486_t	Pathogenesis-related protein bet V I family protein	Medtr8g045570.1	LN633-634	1.59	3.98
MSTRG.86642.2	Calmodulin-binding transcription activator	Medtr8g090205.1	LN635-636	1.07	1.71
138950_t	ABA/WDS induced protein	Medtr6g037220.1	LN637-638	1.86	2.98
172350_t	Rpp4C1 [CC-NBS-LRR resistance protein]	Medtr8g028500.1	LN639-640	3.31	4.56
MSTRG.2242.2	Myb-like transcription factor	Medtr4g086835.1	LN653-654	2.31	3.73
MSTRG.39567.3	WRKY family transcription factor	Medtr1g015140.1	LN655-656	1.77	4.92
MSTRG.26897.3	AP2-like ethylene-responsive transcription factor	Medtr8g068510.1	LN657-658	2.00	1.86
MSTRG.8253.2	Disease resistance protein (TIR-NBS-LRR class), putative	Medtr5g071610.2	LN659-660	4.54	6.38
MSTRG.51124.3	LRR receptor-like kinase family protein	Medtr8g086590.1	LN679-680	-1.05	1.71
141650_t	Nonsense-mediated mRNA decay protein	Medtr5g006990.1	LN641-642	9.14	2.58
MSTRG.79428.1	Transmembrane protein, putative	Medtr3g010283.1	LN643-644	12.65	2.08
MSTRG.8252.1	Disease resistance protein RGA4	Medtr5g070960.1	LN645-646	6.63	2.45
MSTRG.81125.8	Disease resistance protein Rpp4C4, putative	Medtr8g059275.1	LN661-662	6.70	8.58
MSTRG.77395.2	Disease resistance protein (TIR-NBS-LRR class)	Medtr5g092190.1	LN675-676	-5.09	-3.99
**cv. MNGRN-16 vs. cv. Baker nematode-infection**					
MSTRG.40335.1	YABBY-like transcription factor CRABS CLAW-like protein	Medtr5g046230.1	LN651-652	1.53	3.55
174815_t	BZIP transcription factor	Medtr5g015090.1	LN665-666	1.95	1.52
MSTRG.26123.1	MADS-box transcription factor	Medtr7g075870.2	LN673-674	-2.23	-1.57
MSTRG.2692.2	Legume lectin beta domain protein	Medtr1g090973.1	LN681-682	1.67	2.01
MSTRG.72562.4	Glycoside hydrolase family 1 protein	Medtr4g081530.1	LN683-684	5.45	2.53
MSTRG.73426.3	—NA—		LN685-686	1.97	3.93
MSTRG.3948.1	Disease resistance protein (TIR-NBS-LRR class)	Medtr4g015030.1	LN667-668	6.84	9.66
MSTRG.23788.2	F-box-like protein	Medtr1g015450.1	LN669-670	8.57	1.69
**cv. MNGRN-16 control vs. cv. MNGRN-16 nematode-infection**					
9476_t	Polygalacturonase	Medtr6g005630.1	LN627-628	6.34	3.84
31200_t	UDP-glucosyltransferase family protein	Medtr6g042310.1	LN687-688	4.17	2.35
MSTRG.20521.2	LRR receptor-like kinase family protein	Medtr8g086590.1	LN689-690	1.32	1.27
112249_t	Disease resistance protein (CC-NBS-LRR class) family protein	Medtr6g046570.1	LN691-692	4.12	6.01
MSTRG.51124.5	LRR receptor-like kinase family protein	Medtr8g086590.1	LN693-694	1.26	1.22
**cv. Baker control vs. cv. Baker nematode-infection**					
MSTRG.48791.3	Senescence-associated protein, putative	Medtr0055s0050.1	LN695-696	3.92	2.16
MSTRG.64511.2	Receptor-like Serine/Threonine-kinase ALE2-like protein	Medtr4g126270.1	LN699-700	3.27	1.11


To confirm the expression of genes, encoding different nematode effectors identified in this study, qPCR analyses were performed with nine genes in both alfalfa cultivars, using the same mRNA-seq libraries at 3 and 7 DAI. All the genes were validated to be transcribed in both cultivars (Supplementary Figure S3). Overall, our data suggest that *P. penetrans* initially relies on the secretion of a set of the effector proteins to establish infection within both alfalfa cultivars, irrespectively of their level of resistance to the nematode.

## Discussion

Molecular mechanisms of resistance to *P. penetrans* in host plants are still poorly understood. This study presents a first comprehensive assessment of resistant and susceptible interactions of this pathogen with one of the most economically important agricultural crops, alfalfa.

Several important conclusions can be drawn from the findings of this investigation:

(1) Accumulation of TLDs was consistently detected in the root cells of non-infected cv. MNGRN-16 and in the nematode-infected plants of both cultivars. The number of TLDs that accumulated within the cortical root cells in each cultivar was different: deposition of TLDs in the resistant cultivar MNGRN-16 was especially massive, both in control plants, and particularly after infection with *P. penetrans*. Increase in numbers of TLDs in alfalfa root cells during infection with *P. penetrans* and their potential implication in host defenses against RLN were reported as early as 1981 (Townshend and Stobbs, 1981), and later confirmed in 1989 (Townshend et al., 1989). Both studies, however, used alfalfa cultivar Du Puits, that appeared to be susceptible to *P. penetrans* (Olthof, 1982), and did not perform any comparative analyses with alfalfa resistant cultivars. Our data, therefore, not only reassert the increase in TLDs accumulation at the site of nematode infection in alfalfa cultivars contrasting in resistance to *P. penetrans*, but also indicate functional roles of TLDs in constitutive and inducible mechanisms of resistance to this pathogen. In this context, the production of toxic, herbivore-deterrent or -repellent secondary metabolites, typical for many plant defenses strategies, is specifically interesting.
(2) There were no significant differences in the number of nematodes that penetrated resistant and susceptible cultivars at the early stages of infection (up to 7 DAI), suggesting that resistance mechanisms are presumably of post-penetration type and do not prevent nematodes from entering the roots. However, as nematodes feed from the cortical cells, these TLDs possibly play a further role in the course of infection, when host protective responses gradually take effect. Notwithstanding, post-penetration resistance observed in the cv. MNGRN-16 phenotypically and at the subcellular level 4 months after inoculation, likely originated from the early defense signaling initiated by changes in the gene expression levels soon after nematode penetration, and may have been further strengthened due to the constant exposure of nematodes to the resistant environment.
(3) In resistant alfalfa-*P. penetrans* interaction (cv. MNGRN-16), interplay of the following groups of genes representing different signaling and developmental networks can lead to an early recognition of pathogen-associated factors and activation of host defense responses:
  - Cell-wall related genes and protein products as a source of signaling for the activation of defense genes (Narvaez-Vasquez et al., 2005; Bacete et al., 2018).
  - Secondary metabolites, possibly acting as anti-feedants, inhibitors, toxic compounds, precursors to physical defense mechanisms, and antioxidants (Bennett and Wallsgrove, 1994). Differential expression of key genes of the secondary metabolic pathways is consistent with our cell biology observations, thus reinforcing their importance during host–nematode interaction. Critical roles of secondary metabolites in plant resistance against other migratory PPNs (e.g., *Radopholus similis*) have been reported elsewhere (Holscher et al., 2014).
  - Core components of the host immune system: genes encoding nucleotide-binding domain (NBD), leucine-rich repeat (LRR)-containing proteins, and pathogenesis-related proteins.
(4) Constitutive gene expression is critical for the resistance against *P. penetrans*: a number of key genes with defense, recognition, and regulatory functions were highly expressed in the resistant cultivar before nematode infection, making it “primed” for the pathogen attack. Some of these genes were uniquely expressed in the cv. MNGRN-16, emphasizing their possible roles in defense against *P. penetrans*. Pathogen infection did not cause considerable differences in gene expression between the two cultivars, which accentuates the importance of the preformed defenses in the resistant cultivar (vs. pathogen-induced responses). Pre-formed defenses can enable plants to respond more rapidly after exposure to stress (Aranega-Bou et al., 2014). These genetic determinants can also be valuable in new breeding strategies relying on constitutive compounds existing in healthy plants rather than on inactive precursors activated in response to pathogen attack (Osbourn, 1996). Orthologs of the soybean *Rpp4* genes (Medtr8g028500.1 and Medtr8g059275.1), highly induced in cv. MNGRN-16 both prior to infection and during the pathogen attack, along with a few other genes (for example, Medtr5g059140.1 encoding F3H; Medtr4g015030.1, TIR-NBS-LRR; Medtr5g081100.1, subtilisin-like serine endopeptidase; and Medtr1g015450.1, F box-like protein) can be among possible candidates for this approach.
(5) Nematode genes critical for *P. penetrans* development in the host and encoding collagen proteins were down-regulated during infection of the resistant cultivar. Hence, although nematodes were able to penetrate the roots of both cultivars, their further development appeared to be restricted within the cv. MNGRN-16, likely due to the reasons described above. Presumably, this could also happen because of the recognition of nematode effector proteins by specific *R* genes products and activation of downstream effector-triggered immunity (Jones and Dangl, 2006), or due to the absence of recognition proteins in the resistant cultivar, so that genes encoding proteins involved in the interaction with pathogen effectors (susceptibility genes) became inactive (van Schie and Takken, 2014; Kourelis and van der Hoorn, 2018).

**FIGURE 6:**
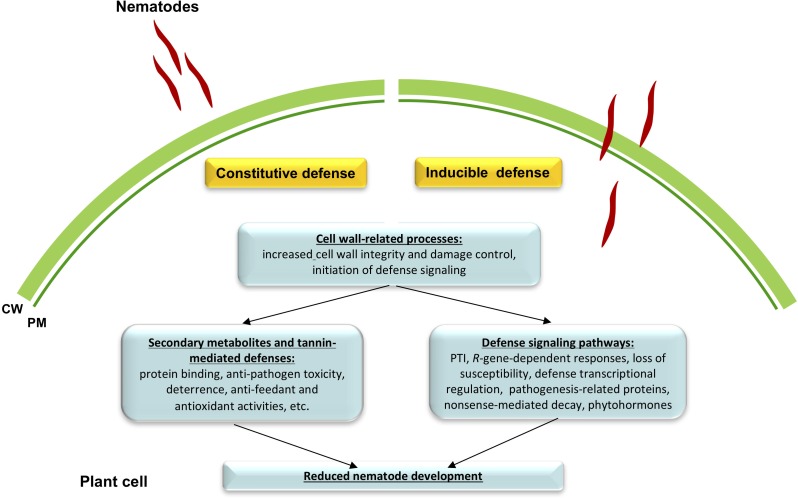
A schematic drawing of defense signaling against root lesion nematode (RLN) *Pratylenchus penetrans* in resistant alfalfa cv. MNGRN-16. Resistant alfalfa interactions with *P. penetrans* depend for the most part on the constitutive defenses that are continuously switched “on” in the plant, in comparison to the susceptible cultivar (e.g., cv. Baker). Once nematode interacts with the plant, a small fraction of genes is activated that together with the preformed defenses is capable of controlling nematode damage.

Altogether, we conclude that resistant alfalfa interactions with *P. penetrans* depend, for the most part, on the constitutive defenses that are continuously switched “on” in the plant, while inducible defenses generated during infection, might have a lesser, or at least more specific role in the establishment of resistance. It also appears that in general the same pathways participate in constitutive and inducible defense reactions against *P. penetrans*, although their intensity level and final gene products are likely different. Tentative mechanisms of the resistance pathways in cv. MNGRN-16 are summarized in Figure 6.

It is also worth noting that alfalfa as a species may implement unique resistant pathways against PPNs, dissimilar to other plants. For instance, alfalfa resistance to *M. incognita*, a sedentary PPN from a different family (Meloidogynidae) is not defined by a localized hypersensitive response near the feeding site, a reaction controlled by *R* genes and observed in other species (Potenza et al., 2001; Dhandaydham et al., 2008; Postnikova et al., 2015). Our earlier study on alfalfa interaction with *M. incognita* has suggested that defense strategies other than *R* gene-based responses, such as elevated levels of basal or constitutive gene expression under control conditions, might be involved in the host resistance pathways (Postnikova et al., 2015). Likewise, no HR against migratory nematode *P. penetrans* was observed in the current study, although alfalfa resistance to *P. penetrans*, presumably reliant on constitutive defenses, appears to be different from the plant’s responses to other PPNs. In particular, the induction of host genes involved in biosynthesis of secondary metabolites, especially in phenylpropanoid pathway, and accumulation of secondary metabolites in the cells seem to be critical for alfalfa resistance to *P. penetrans*. This could provide valuable insights into the development of alternative strategies to control RLNs. Notwithstanding, a hypothesis of alfalfa’s atypical mechanisms of resistance to PPNs requires further investigation.

## Data Availability

Raw sequencing reads have been deposited in the NCBI Sequence Read Archive (SRA) under the BioProject ID PRJNA547347 and BioSamples Accession Nos. SAMN11963269 to SAMN11963288.

## Author Contributions

PV and LN designed, executed, supervised the project, analyzed the data, and wrote the manuscript. JM performed the transmission electron microscopy. JS performed the bioinformatic analyses. JE contributed to the manuscript revision. All authors read and approved the final manuscript.

## Conflict of Interest Statement

The authors declare that the research was conducted in the absence of any commercial or financial relationships that could be construed as a potential conflict of interest.

## Footnotes

1 http://alfalfa.org/newsletter/180327nafanews.htm

2 http://www.medicagohapmap.org/home/view

3 http://exon.gatech.edu/GeneMark/

4 https://ccb.jhu.edu/software/stringtie/gffcompare.shtml

5 https://www.ncbi.nlm.nih.gov/

6 https://jgi.doe.gov/data-and-tools/bbtools/bb-tools-user-guide/bbduk-guide/

7 http://docs.blast2go.com/user-manual/

8 http://bioinfo.cau.edu.cn/agriGO/analysis.php

9 http://planttfdb.cbi.pku.edu.cn

10 https://www.idtdna.com/scitools/Applications/RealTimePCR/
