# Supplementary material for: Cellular and Transcriptional Responses of Resistant and Susceptible Cultivars of Alfalfa to the Root Lesion Nematode, Pratylenchus penetrans
Source: Front Plant Sci. 2019 Jul 31;10:971. doi: 10.3389/fpls.2019.00971 (PMC6685140; doi:10.3389/fpls.2019.00971)
Supplement: Supplementary file 7 [file Data_Sheet_1.docx]

**Supplementary information for following article:**

**Resistant and Susceptible Responses to Root Lesion Nematode (*Pratylenchus penetrans*) in Alfalfa**

**Paulo Vieira^1,2^, Joseph Mowery^3^, Jonathan D. Eisenback^2^**, **Jonathan Shao^1^ and Lev G. Nemchinov^1^**

^1^ USDA-ARS, Molecular Plant Pathology Laboratory, Beltsville, MD 20705-2350, USA, ^2^ School of Plant and Environmental Science, Virginia Tech, Blacksburg, VA, 24061, USA, ^3^ USDA/ARS, Electron and Confocal Microscopy Unit, Beltsville, MD 20705-2350, USA

**Correspondence:** Lev Nemchinov (lev.nemchinov@ars.usda.gov)

**Figure S1.** Total number of nematodes associated per individual plant of cv. Baker and cv. MNGNR-16 at 7 days after nematode infection. Nematode-infected roots of both cultivars were stained with acid fuchsin, and the total number of nematodes penetrated the roots was compared among individual plants. Four independent biological experiments were performed using a minimum of 10 plants per cultivar.

**Figure S2.** GO annotation of nematode DETs in cv. Baker and cv. MNGNR-16, respectively.

**Figure S3.** Validation of *Pratylenchus penetrans* effector genes by RT-qPCR. Expression profiles of nine *P. penetrans* effectors were determined at 3 and 7 days after nematode infection (DAI), using three independent biological experiments. Values are given relative to *18S* rDNA reference gene.
